# Mr. Bradley's *Cases in Surgery, &c.*

**Published:** 1801-03

**Authors:** John Pearson

**Affiliations:** Surgeon, Golden Square.


### Mr. Bradley's *Cases in Surgery, &c.* Communicated by Mr. John Pearson, Surgeon, Golden Square.

### *A Case of Abscess in the Groin.*

ELIZABETH MARSDEN, an unmarried woman, aged 33 years at the time of her death, was, in the early part of November, 1786, feized with pain and heat in the region of the ftomach, attended with a fmall and quick pulfe, thirft, alternate rigors and heats, with other indications of internal inflammation. In confequence of a difpofition to vomit, fifteen grains of the pulv. ipecac. with two drachms of the antimonial wine, were given in a fmall draught, which puked her three times; after this, feventy grains of *Fordyce's prophylactic powder* were given, which procured her three or four motions. The faline mixture was alfo ordered, and a blifter applied to the ftomach; thefe remedies relieved her for that time; but having occafion to remove to a diftant part of the country, fhe foon after grew worfe, and, in confequence, applied to other practitioners.

After an abfence of fourteen months, fhe returned with a large circumfcribed fulnefs, or tumour, occupying the part previoufly affected, attended with pain and uneafinefs, efpecially on preffure, but without difcolouration, or any evident figns of containing matter: A flight hectic was prefent, which

 feemed feemed to have confiderably affected her health. Five months after this fhe was feized with *cholera morbus*, which, after continuing violent four or five days, ceafed, but ever afterwards returned, though generally in a lefs immoderate degree, three or four times, or oftener, in a year, till the time of her death. This firft attack produced a very confiderable effect in reducing the tumour, and the remaining fulnefs was entirely removed by the fubfequent evacuations; thefe generally weakened her much, but foon afterwards fhe regained a tolerable portion of health.

In the latter part of the year 1788, fhe had fymptoms of a crural hernia, for which a fteel trufs was applied, and worn for fome time, (but how long I could never learn) till, imagining herfelf well, it was, as is too often the cafe, difcontinued.

In the beginning of May, 1794, and fome time fubfequent to the ufe of her trufs, fhe had again fymptoms of her hernia, which, in a month or fix weeks, had protruded as formerly. On the 6th of July following, fhe was feized with a confiderable vomiting, and at the fame time had a profufe ftool; thefe evacuations were fucceeded by pain in the hernia and lower part of the abdomen, which continued diftreffing till the 8th, when I found her feverifh, having a fmall and quick pulfe, confiderable thirft, frequent rigors, alternated with heats and pain, and difficulty in making water, and which was voided in fmall quantities: She had not had a ftool fince the 6th, nor vomited for the laft twenty hours. The tumour in the groin was more painful and diffufed than thofe generally arifing fimply from hernia; and on making a gentle preffure upon it, its contents returned into the abdomen, leaving an unufual fulnefs of the integuments.

An ounce of the common cathartic falts was diffolved in fix ounces of boiling water, and a table fpoonful of this folution was ordered to be taken every hour, till the body fhould be well opened: A cooling diet, fuch as tea, coffee, with dry toaft, and now and then a little thin chicken broth, was recommended; balm tea and barley water were ordered to be drank plentifully, and a fupine pofture was alfo ftrictly enjoined. The aq. veget. miner. was applied to the fwelling; but it not only occafioned moft excruciating pain in the bowels, but even fometimes vomiting.

9th. The medicine had operated three times, and was ordered, along with the lotion, to be difcontinued; the hernia was apparently down, and was reduced as before, but the operation gave great pain; the integuments were flightly difcoloured, and an evident fluctuation of matter was perceived.

By

By reafon of thefe circumftances, a bread and milk poultice was applied warm to the tumour every fix hours, and warm water and fweet oil were frequently injected into the rectum; the inflammatory fymptoms continued increafing, although fhe perfpired to day for fome time very freely, but without affording any mitigation of her pain; now become very acute in the bowels and lower part of the abdomen.

10th. She was extremely reftlefs in the night, and had no fleep, though fhe took fifteen drops of tinct. opii every hour for three times; at noon this day her fever run very high, and the pain in the forementioned places was very acute and diftreffing; fhe was feized with a moft violent rigor, fucceeded by great heat and perfpiration; difcolouration and tenfion now extended over the whole right hypogaftric region, and the tumour was daily increafing in magnitude: the poultice was continued, but the glyfter omitted, as it feemed to afford no relief.

11th. She took no opiate laft night, and refted very indifferently, by reafon of pain; the fever this morning ran very high, and the abfcefs had increafed confiderably, and on the top of which appeared a flight livid tinge. Ordered the glyfter to be again repeated, with the addition of a little Caftor oil, as fhe had not had a ftool fince the morning of the 9th; this, however, was objected to, but the treatment, in all other refpects, was continued.

12th. Took twenty drops of tinct. opii at three feparate times laft night, which procured two hours of difturbed reft, during which fhe perfpired very freely. A veficle now appeared on the apex of the tumour, with lividnefs to the extent of a fhilling; her pulfe was quick, but fmaller and more enfeebled, yet her thirft confiderable: the rigors and heats were more frequent, and lefs violent, and were fucceeded by more partial perfpirations. Her pain was lefs immoderate, but yet confiderable, and the diftrefs in making water not much alleviated. Had yet had no ftool, and ftill objected to the adminiftration of a glyfter.

Ordered two table fpoons full of the *decoct. cort. Peruv.* every three hours, taking at the fame time fifteen grains of the kali ppt. diffolved in two ounces of water, and as much of the *acid vitriol. dilut.* as was fufficient for faturating the fame.

13th. Slept about two hours in the night without the aid of an opiate; but was attacked with a cold and hot fit this morning, fimilar to the paroxyfm of a quartan ague; the fhivering lafted upwards of an hour, and the fucceeding heat and perfpiration were proportionally large: the pulfe was weak and very quick, being about 134 in a minute, and her ftrength was confiderably reduced, yet her fpirits were tolerably good; the the gangrene increafed, though the pain was ftill confiderable. A fuppofitory of treacle, with the addition of a little butter and falt, was adminiftered, which brought away a fmall quantity of hardened fæces.

14th. Slept four hours laft night without an anodyne, and the fuppofitory was again adminiftered, with nearly the fame effects as yefterday: The febrile paroxyfm came on, obferving nearly the fame time and degree of violence as before; had partial and clammy fweats during the intervals, notwithftanding a confiderable degree of arterial action ftill exifted. An ounce of compound tincture of bark was added to her decoction, and which was ordered to be taken as before. Four or five fmall wine glaffes of red Port were alfo ordered to be taken in the courfe of twenty-four hours, and a more nutritious diet was recommended, fuch as good broths, or even animal food, if the ftomach could take it. Lint dipped in equal parts of ol. terebinth. and *bals. capaivi* was applied to the gangrenous part, previous to the application of the poultice; now changed to one compofed of oatmeal and brifk ale, with the addition of a little fweet oil.

15th. Slept two or three hours laft night, and in other refpects appeared the fame as yefterday: the paroxyfm came on three hours earlier than the laft, and was, in violence and duration, fimilar to the preceding. Had a natural ftool in the afternoon, and the gangrene appeared now ftationary, and fomewhat offenfive.

16th. The paroxyfm laft night returned three quarters of an hour earlier, but in other refpects fimilar to the laft: her pulfe were now ftronger, and fomewhat flower, and the intermediate and partial fweatings more inconfiderable; had yet fome pain, though far lefs immoderate than formerly; flept very little laft night, but to day dofed a good deal: the gangrene was feparating, and the treatment continued as before.

17th. The acceffion of the fit laft night was an hour and a half earlier than, but in other refpects nearly alike to, that immediately preceding it: the intermediate fever this day ran higher than ufual; the ufual fuppofitory was adminiftered, but without effect. On removing the poultice this evening, fome of the gangrenous part floughed off, which was followed by a quantity of putrid fanies; after this, rufhed out about feven or eight ounces of well concocted matter. The fame treatment was, however, continued.

18th. Slept tolerably laft night; and the feverifh paroxyfm came on two hours and a half later than the laft, and was lefs fevere than any of the preceding. On removing the poultice this morning, a quantity of fæces was difcovered; the fame circumftance circumſtance was again obſerved in the evening, together with a conſiderable diſcharge of matter.

19th. Slept three hours comfortably laſt night, and to day had ſome ſhort intervals of repoſe: the paroxyſm was earlier by a quarter of an hour than the laſt; ate ſome currant pudding and broth to dinner: In the evening, at dreſſing, had a large diſcharge of fæces and matter from the opening, and the whole of the mortified part this day ſloughed off, leaving an ulcer four inches and a half in length, and nearly three inches in breadth, and which extended from below the *os pubis* upwards toward the ſpine of the *os ilium.* Dreſſed this evening with dry lint, and a pledget of *ung. de sperm. ceti*; and the bark, along with a milk diet, was alone preſcribed.

She henceforth gradually grew better, both with regard to the ulcer, and her health in general, till the 8th of Auguſt, when ſhe again vomited a large quantity of chocolate coloured fluid, attended with pain in the bowels, and an exacerbation of her hectic ſymptoms, all which were relieved by an increaſed diſcharge from the ulcer. This evacuation in a day or two weakened her conſiderably; but by diſcontinuing the uſe of the bark, and taking the chalk mixture, it was checked.

Hence, to the time of her death, ſhe was variouſly affected; ſometimes ate well, was cheerful, and tolerably free from pain; and her hectic ſymptoms almoſt diſappeared for four or five days, ſometimes a week or ten days, or even longer, when ſhe was ſuddenly ſeized with pain, and frequently ſwelling of the abdomen, increaſe of fever, and ſometimes vomiting, though not ſo often as formerly; theſe were terminated by a large diſcharge of moſtly a thin, acrid, and browniſh fluid from the ulcer.

The ſevere paroxyſms which took place during the time that matter was pent up in the abſceſs, and which aſſumed the intermittent type, particularly when a change was effected in the tumour, and the ſyſtem at large, are circumſtances worth remarking, though doubtful whether affording any hint towards elucidating the nature of intermittents in general.

The diſcharge from the ulcer varied, not only in quantity but in colour and conſiſtence; when the moſt profuſe, it was thin, and chocolate coloured; and when leſs immoderate, aſſumed a more pus-like appearance. At all times, however, but more eſpecially when copious, it was ſo acrid as to produce much excoriation on the contiguous parts: Cleanlineſs, and the application of ſaturnine preparations, with the forementioned dreſſings, were frequently and occaſionally made uſe of. The fæces evacuated from the ulcer generally when unmixed with any other diſcharge, were whiter, and of a leſs conſolidated conſiſtence confiftence than thofe voided the natural way; and for fome time fubfequent to the rupture of the abfcefs, animal food was voided by the ulcer, apparently in a half digefted ftate. The pain and fwelling of the belly, accompanied with a fpafmodic ftate of the abdominal mufcles, were frequently diftreffing, and generally preceded a liquid and increafed difcharge from the ulcer; to obviate which, opiates occafionally were had recourfe to.

Her pulfe, from the 8th of July to the time of her death, was generally from 100 to 125; and as Nature funk, it became fmall and contracted; infomuch, that fix weeks previous to her death, it was fcarcely diftinguifhable; notwithftanding, all this time fhe was perfectly rational, and ftrongly influenced for the laft week with the idea of dying immediately, provided fhe were raifed up from a fupine to an erect pofture. At her own preffing folicitation the experiment was made, and fhe died almoft inftantly.

Her tongue, at the beginning of the diforder, reckoning from the formation of the abfcefs, was generally furred, and fomewhat yellowifh, efpecially in the middle and back part of it; but in the latter ftage, was clean, and of a raw appearance.

The urine, during the whole time, was moftly high coloured, and depofited a ftrong purulent fediment, with a lateritious tinge; fometimes, however, and efpecially towards the laft, it was paler, with, and even without, a dark coloured nubicula.

About the middle of her ficknefs fhe had a troublefome cough, with fome expectoration of *mucus*, and attended with fome pains in the cheft.

From the fourth day fubfequent to the breaking of the abfcefs, fhe had evacuations *per anum*, fometimes every day, but oftener every fecond or third day. Thefe, for the firft month, or five weeks, were in a very trifling degree; but, either from the effects of oil and water gruel, daily thrown up into the *rectum*, or more probably from the efforts of Nature, an increafed quantity of fæces was gradually obtained; always, however, bearing marks of having paffed fome frictured part of the inteftine.

She often complained during her illnefs of pain in the infide of the thigh, knee, and leg of the fide affected, all of which were confiderably emaciated.

She menftruated in a fmall degree on the 18th of September, being the only time fubfequent to the formation of the abfcefs in the groin; but previoufly fhe was regular.

For fome time before her death, all the different excretions of the body were confiderably diminifhed, and the fæces difcharged charged from the ulcer were in a liquid ftate; but thofe evacuated by the *anus* were generally of a hardened confiftence. The ulcer, from the firft, healed very expeditioufly; and long before her death was reduced to two fmall openings, about half an inch afunder, and little more than capable of admitting a moderate fized garden pea.

On opening the body after death, which happened on the 28th of November, 1794, the mefenteric glands were the parts that chiefly exhibited marks of difeafe, efpecially fome that were fituated behind the *duodenum*, and contiguous to the back; thefe were indurated, and contained a cheefe-like fubftance, and a few of them were as large as a fmall chefnut. The reft of the mefentery feemed difeafed; and that part connecting the inteftines together was thickened, and fo much fhrivelled up, as to give the latter a very dilated appearance. In the mefentery of the loins, two or three inches below the right kidney, was found an abfcefs, containing about two ounces of thin purulent matter. The ftomach and bowels exhibited no preternatural appearance, except the blood veffels of the fmaller inteftines in particular were confiderably enlarged. The liver and gall bladder were free from any marks of difeafe; but the membranous covering of the former was much thickened, and adhered firmly to the diaphragm: The fpleen and pancreas alfo appeared found.

On examining the aperture in the groin, it was found that a portion of the inteftine *ileum* adhered firmly to Poupart's ligament, and was puckered, thickened, and occupied about the fpace of a large halfpenny. From this part iffued two openings externally, the one above and the other below this ligament; the larger of thefe was little more than fufficient to admit the introduction of a female catheter. The paffage which led forwards to the under part of the inteftinal canal was fo narrow, that it was with fome difficulty I paft the firft joint of my little finger through it: The peritonæum contiguous to this adhefion, for fome diftance, was extremely thickened, and even that portion of it in contact with the bladder, as well as the fundus of that vifcus, partook of this enlargement. The parts of generation were found, and the pfoas mufcle free from difeafe.

One inference to be drawn from this cafe, and which alone can recommend it for publication is, to warn us againft fuffering fimilar abfceffes to break of themfelves, as the confequences are not only extreme pain and diftrefs from the matter being pent up beneath a ftrong aponeurotic and membranous covering, but a very confiderable lofs of fubftance, with an increafe of hectic, and a large aperture for the admiffion of air into into the cavity of the abdomen already diſeaſed. Theſe evils might, in ſome meaſure, be obviated by a timely opening; for although no permanent advantage would accrue from it, yet the alleviation of pain and diſtreſs, and the probable procraſtination of death, objects which probably would be the reſult, and which with the humane and rational ſurgeon are always *deſiderata* of moment.

In this kind of abſceſs, which is complicated with hernia, an apprehenſion may prevail againſt the uſe of the knife, which, if employed, muſt inevitably wound the hernial contents, (for there was little doubt here of the matter being confined within the inteſtine;) but could even an imprudent uſe of this inſtrument be productive of worſe conſequences than thoſe reſulting from ſpontaneous rupturing? As to the manner of opening theſe tumours, a large orifice ſeems inadmiſſible from the greater expoſure of the cavity of the abdomen to the external air; perhaps the moſt eligible method will be to thruſt an abſceſs lancet gently forwards, or if any doubt be entertained of the matter being confined within the inteſtine, diſſecting carefully through the external covering, till matter be found, may be the moſt adviſable. It may probably be found, that the moſt proper time for performing this operation is when the firſt ſymptoms of lividneſs appear; for I have generally obſerved, that a conſiderable degree of excitement, in the formation of abſceſſes, tends not only to expedite the cure, after their contents are evacuated, but has very ſalutary effects on the ſyſtem at large.

### *A Caſe of Tertian Intermittent, &c.*

Sarah Priest, a young girl of fourteen years of age, and of an athletic conſtitution, and who had never menſtruated, was ſeized with the paroxyſm of a tertian ague, Jan. 14, 1795. The cold ſtage continued upwards of an hour, and was ſucceeded as uſual by a hot fit, which laſted rather more than two hours, and then ſubſided without any evacuation by the ſkin.

Jan. 16. The paroxyſm returned to day about the ſame time, but was ſomewhat longer in duration.

17th. To day being the firſt time of ſeeing her, I found her labouring under conſiderable fever, pulſe quick, tongue white and ſomewhat parched, ſome pain in the head and loins, and a diſpoſition to nauſea and coſtiveneſs; to remove which, fifteen grains of the pulv. ipecac. with one grain of the tartar emetic were given, which puked her ſeveral times. After this one drachm of *Fordyce's prophylactic powder* was ordered, which procured four ſtools. Eight grains of *nitre* with the ſame quantity quantity of *white ſugar*, were next preſcribed to be taken every three hours, along with three table ſpoons full of the *ſaline mixture*, eight ounces of which contained four ſcruples of the *kali*, with as much lemon juice as was ſufficient to ſaturate the ſame.

18th. The fit came on four hours earlier than uſual, and was ſimilar to the laſt in point of violence and duration, but yet terminated by no perſpiration. In other reſpects ſhe was the ſame as before.

19th. Little alteration ſince yeſterday, the intermediate fever however appeared ſomewhat leſſened.

20th. The paroxyſm came on ha'f an hour ſooner than the laſt, and was of equal ſeverity and duration, and yet unſucceeded by any diſcharge from the ſkin. As the intermediate and febrile ſymptoms were now conſiderably abated, the following was ordered:

℞. Pulv. cort. Peruv. ʒiij. Kali pp. ʒſs. Aq. nuc. moſch. ℥ſs. Aq. font. ℥vij. M.

Two table ſpoons full of this mixture were taken every three hours.

21ſt. Was better to day than ſhe hitherto had been during the intervals of the fits, being now pretty cool without thirſt or pain, and her appetite was conſiderably better.

22d. This day ſhe had no return of paroxyſm, and in all other reſpects ſeemed very well. Had diſcontinued her mixture laſt night, after having only taken eight ounces of it, and ſhe could not be perſuaded to take it afterwards.

24th. At or about the time of the laſt fit, which was in the night, the patient, according to her own account, was ſeized with pain in the head, uneaſineſs and oppreſſion at the ſtomach, conſiderable thirſt and heat, but without rigors; theſe, on abating, were ſucceeded by ſleep, and a hæmorrhage, as was ſuppoſed, from the mouth. Six hours afterwards petechiæ were diſcovered on the arms, ſhoulders, and upper part of the trunk, and even on the legs, but the ſpots on the laſt were ſmall and innumerable. On looking into the mouth, a number of petechiæ of a gangrenous aſpect appeared within the lips, on the inſide of the cheeks and fauces. Her pulſe was quick, but not weak, and ſhe had ſome thirſt, with liſtneſſneſs and proſtration of ſtrength. Had no ſickneſs or nauſea, and was regular as to ſtools, which exhibited no unnatural appearance. Bled none this day ſince ſhe awoke in the morning, but her ſaliva was now and then ſlightly tinged.

Ordered three large table ſpoons full of a ſtrong decoction of bark, ſix drachms of which were boiled down in twelve ounces of water to ſeven, and an ounce of the compound tincture added to it, and along with which were taken twelve drops of the elix. *vitriol. acid.* Half a pint of red port wine made into *negus*, and acidulated with lemon-juice, was ordered to be taken in the courſe of the day; and a nouriſhing diet, ſuch as broths, jellies, &c. was alſo recommended.

25th. Bled in the night as before, but without being ſenſible of any previous indiſpoſition. As the hæmorrhage happened in her ſleep, neither its acceſſion, duration, or violence, could be aſcertained. In other reſpects ſhe was as the day before.

26th. Had bled largely as uſual from the mouth, in the night during ſleep. Her legs were now ſwelled, and livid, and the petechiæ were much increaſed, eſpecially on the face, ſhoulders, and breaſt. The ſpots within the mouth had alſo a more gangrenous aſpect, but the reſt of the mouth had an uncommonly pallid appearance. Her pulſe was about one hundred, and weak, and her thirſt was yet conſiderable, though her tongue was moiſt and clean. She was liſtleſs, low ſpirited, and free from pain, and was alſo regular as to ſtools, which were of a natural conſiſtence, but ſomewhat more than uſually offenſive, and dark coloured.

The ſtrength of her mixture was increaſed from ſix drachms of the bark to one ounce, the ſame quantity of the decoction to be taken as before, along with fifteen drops of the elixir, inſtead of twelve. Her wine was alſo ordered to be increaſed to a pint a day.

27th. Much the ſame as yeſterday, and had bled as uſual in the night, whilſt aſleep Inſtead of the compound tincture of bark, an ounce of the tincture of *catechu* was added. Milk and rice were now taken along with her former diet.

28th. Bled as uſual laſt night, and complained of great ſtiffneſs in her hams and knees, and her legs were much ſwelled, and more livid and œdematous. The petechiæ were more univerſal, and her breath offenſive. She complained of ſome ſickneſs, and was greatly dejected. She was delirious the fore part of the night, but afterwards ſlept tolerably. Inſtead of the vitriolic, twelve drops of the muriatic acid were preſcribed to be taken in the ſame manner as the former; and in the intervals of taking her medicine, lemon-juice in different vehicles was plentifully drunk.

29th. This day ſhe went a few yards out of doors, and on her return was ſeized with rigors, and conſiderable pain in the back and lower extremities, eſpecially the right leg, which now, in addition to lividneſs, put on ſymptoms of inflammation, and was greatly ſwelled. Two or three large *vibices* appeared in different parts of the body, one of which ſurrounded the right right eye, and occupied the fpace of the *orbicularis* mufcle. Her pulfe was increafed to one hundred and fix, yet her thirft much the fame; her petechiæ however bore rather a more favourable afpect, and fhe bled only about half the ufual quantity in the night.

30th. Better in all refpects, as the pain in the loins and legs had vanifhed; and the appearances of the latter, with regard to the colour and fwelling, much more favourable. The petechiæ alfo were changing from a livid to a chocolate colour, and her pulfe came down to ninety, and her urine was turbid and fimilar to new beer mixed with yeaft. Had no hæmorrhage laft night, and has been without ftool for the two laft days; but on ordering the *tinct. catechu* to be omitted, fhe had a motion the fame evening. Her appetite now increafed, and the fame plan was ordered to be purfued.

31ft. She continued in all refpects to recover. The petechiæ were either daily difperfing or becoming frefher, and the fwelling of her legs was confiderably reduced, and the lividnefs had nearly difappeared, notwithftanding which fhe bled a few drops in the night. Her pulfe was now at eighty-feven, and as fhe complained of being tired of her medicines, the dofes were not only leffened to two table fpoons full of the mixture, and eight drops of the acid, but ordered to be taken only three or four times a day. Thefe fhe could only be perfuaded to take two or three days further, but her pint of wine fhe daily continued taking a week longer.

On the 5th of February, fcarcely a veftige of petechiæ was difcernible, and fhe had been free from any further attacks of hæmorrhage, and in all other refpects appeared well. She henceforth rapidly regained her former health, which fhe has continued to enjoy to the prefent time.

This girl, previous to this complaint, had always enjoyed an uninterrupted ftate of health from her infancy, and had been accuftomed to a good deal of bodily exercife, efpecially within doors. Her diet had ufually confifted of milk, and fometimes malt liquor as a fubftitute, farinaceous, and occafionally animal food, partly frefh and partly falted. Though apparently of a found conftitution, and without any external mark of fcrophula, yet her parents were deeply tainted with that diforder.

After the firft and fecond attack of hæmorrhage, fome proper attendants were appointed to obferve the time of acceffion, and every other circumftance, with regard to any fubfequent bleeding that might enfue. The refult of thefe obfervations were, that the third attack came on at two o'clock in the morning, after the patient had been unremittingly afleep for four hours, hours, and although, previoufly, fhe had been fomewhat reftlefs, with apparently increafed heat and flufhing of the face, yet when the bleeding came on fhe appeared comfortable, ftill, and laid on her fide. After bleeding about five and thirty minutes, *per stillicidium*,* fhe awoke, and then it ceafed. The fourth attack came on half an hour later than the preceding, and was ufhered in by lefs reftleffnefs and apparent heat. After bleeding nearly forty minutes it ceafed, and fhe continued fleeping forwards for an hour, and then awoke and fell afleep a fecond time, but without having any further return of the hæmorrhage. On the fifth night, fhe was awoke at two o'clock, and kept from falling afleep again till half paft three; and then yielding to the power of fleep, ten minutes afterwards the hæmorrhage returned, without any previous indications, and was more profufe than before, but of fhorter duration, as it only continued about twenty-eight minutes, though the faliva for feveral hours afterwards was flightly tinged. With refpect to the fixth attack of hæmorrhage, from the careleffnefs of the attendants no proper account can be given.

From a number of collated circumftances, attendant both on the two firft and fubfequent hæmorrhages, I am of opinion that they returned at nearly regular periods; for notwithftanding on the fifth morning, the bleeding was later in its return, yet probably its procraftination was lengthened by keeping her awake beyond the former periods of attack, for, about an hour previous to its acceffion, it was with fome difficulty fhe was kept from fleeping. This circumftance joined to the confideration of the hæmorrhage always commencing during fleep, may in fome meafure account for its procraftination on the fifth morning.

The urine throughout her diforder was changeable, both in quantity and appearance. During the firft and intermittent ftage, it was in large quantity and of a natural colour, efpecially after the paroxyfm, and depofited a fmall fediment, partly flocculent and partly purulent; but during the intervals, was fomewhat higher coloured, and generally without fediment. In the latter ftage it was generally paler than natural, and the fettlement largely and folely flocculent. On the 30th, the day after catching cold, the urine however put on a confiderable change, fuch as is defcribed in the narrative of that day.

She

* The quantity of blood loft could not be afcertained. I fuppofed about an ounce and a half at a time, therefore the bleeding muft have been very flow.

She never appeared to have any rigors from the cure of the intermittent fever to the 29th, being the day of catching cold, nor any increafed heats in that interval, except thofe already defcribed as ufhering in the hæmorrhage; and as to perfpiration, not the leaft was perceptible during her illnefs, but on the contrary the fkin always felt hot and dry.

The weather, for feveral months prior to the firft acceffion of this complaint, had been exceedingly changeable. In the autumn there was much rain, which continued into December; to this fucceeded alternate frofts and thaws, with almoft daily changes of the wind. A fevere ftorm of froft and fnow, at length fet in the beginning of January, and with the exception of one or two intermiffions of flight thaws, continued to the commencement of her diforder.

Newfolme, the place of her refidence, is a fmall village fituated rather in a mountainous part of the country, on the middle of a declivity of confiderable extent. It is expofed much to the north and north-weft winds, and I have thought its inhabitants unufually fubject to feverifh complaints; and befides, I once met with another cafe of tertian in this place, which was a girl of about the fame age and temperament, and who had never menftruated. The diforder was idiopathic, and unattended with any anomalous fymptoms. The paroxyfms returned at equal intervals, and were terminated by copious perfpiration, and eafily gave way to the bark.

During a practice of almoft twenty years in this town and neighbourhood, I never faw an intermittent as a primary affection, a native of the place, excepting thefe two cafes; yet, notwithftanding this, many people formerly, who were in the yearly habit of repairing to the eaftern part of this county as reapers, imported this diforder on their return, and which generally continued with them throughout the winter, but either left them fpontaneoufly on the return of warm weather, or eafily yielded to the bark. Latterly, however, this complaint is far lefs frequently imported than formerly, a circumftance arifing either from the harvefts being fomewhat earlier, or what is more probable, the country being more cleared and better drained.

A great proportion of thefe intermittents were tertian, and the unhappy fufferers labouring under them generally were taking divers remedies or noftrums throughout the winter, and at the fame time imprudently expofing themfelves to the cold. For inftance, the bark they would take for three or four days, till imagining themfelves well, they then would generally difcontinue its ufe, and confequently incur a relapfe, againft which this remedy, or fome other, was again had recourfe to, with with ufually no better fuccefs than before. Thus were thofe unhappy people haraffed throughout the winter; and even on the return of warm weather, were with more difficulty cured than thofe whofe complaint had continued uninterruptedly throughout the winter, and for which no remedies had been taken; and befides, I have thought that affections of the liver, dropfies, &c. were more apt to fucceed to the former clafs of patients than the latter.

[ To be continued. ]

